# Large-scale spatial variation of chronic stress signals in moose

**DOI:** 10.1371/journal.pone.0225990

**Published:** 2020-01-13

**Authors:** Göran Spong, Nicholas P. Gould, Ellinor Sahlén, Joris P. G. M. Cromsigt, Jonas Kindberg, Christopher S. DePerno

**Affiliations:** 1 Department of Wildlife, Fish, and Environmental Studies, Swedish University of Agricultural Sciences, Umeå, Sweden; 2 Department of Forestry and Environmental Resources, Fisheries, Wildlife and Conservation Biology Program, Raleigh, NC, United States of America; 3 Department of Zoology, Centre for African Conservation Ecology, Nelson Mandela Metropolitan University, Port Elizabeth, South Africa; 4 Norwegian Institute for Nature Research, Trondheim, Norway; Universitat Autonoma de Barcelona, SPAIN

## Abstract

The physiological effects of short-term stress responses typically lead to increased individual survival as it prepares the body for fight or flight through catabolic reactions in the body. These physiological effects trade off against growth, immunocompetence, reproduction, and even long-term survival. Chronic stress may thus reduce individual and population performance, with direct implications for the management and conservation of wildlife populations. Yet, relatively little is known about how chronic stress levels vary across wild populations and factors contributing to increased chronic stress levels. One method to measure long-term stress in mammals is to quantify slowly incorporated stress hormone (cortisol) in hair, which most likely reflect a long-term average of the stress responses. In this study, we sampled 237 harvested moose *Alces alces* across Sweden to determine the relative effect of landscape variables and disturbances on moose hair cortisol levels. We used linear model combinations and Akaike’s Information Criterion (corrected for small sample sizes), and included variables related to human disturbance, ungulate competition, large carnivore density, and ambient temperature to estimate the covariates that best explained the variance in stress levels in moose. The most important variables explaining the variation in hair cortisol levels in moose were the long-term average temperature sum in the area moose lived and the distance to occupied wolf territory; higher hair cortisol levels were detected where temperatures were higher and closer to occupied wolf territories, respectively.

## Introduction

Short-term stress allows individuals to perform better in emergency situations (e.g., imminent threat of predation or physical conflict) whereas, long-term or chronic stress affects individual fitness negatively [1, 2], with potential implications for the performance of wild populations. Further, the physiological consequences of chronic stress include reduced fertility [3], impaired cognition [4], weaker immune system [5], lower body condition and survival [6]. Despite this overarching importance of chronic stress for individual and population performance, little is known about factors affecting chronic stress and its distribution in wild populations.

Chronic stress may be expressed in a population as increased disease or decreasing population growth [7], but these trends may be masked by intense harvest or mistaken for density dependent processes. Because changes in underlying vital rates can have direct effects on population dynamics and viability, disentangling the role of chronic stress for vital rates in wild populations is important and particularly true for species with slow life history or small populations. Furtherthere is often a time lag between disturbance events and the associated population decline, where the actual population stressors are often masked or missed. Hence, real-time data to monitor chronic stress levels could provide an early warning system of changes that affect populations [8].

Across a variety of species, stress levels and individual health are negatively affected by multiple factors. These factors include fasting [9]; habitat fragmentation [10]; anthropogenic activities (e.g., roads, railways, oil and gas well-sites, cut-lines, power-lines, pipelines, and forest harvest blocks, [8]), disease, injuries, discomfort, or pain [11]; climatic shifts and heat [12, 13]; predation risk [1, 14]; competition [15]; mating competition [16, 17] and displacement [18]. For example, [13] noted that polar bears *Ursus maritimus* were under higher levels of physiological stress during years with less ice cover and less access to seals, and [1] noted that predation risk accounted for chronic stress and deterioration of reproduction in snowshoe hares *Lepus americanus*. Notably, there may be synergistic effects of stressors occurring across the landscape, and the frequency and magnitude of these stressors may determine the ultimate allostatic load (i.e., the physiological consequences of long-lasting exposure to repeated or chronic stress) on an individual or population.

Cortisol is a hormone involved in a wide range of physiological processes such as immunity, digestion, reproduction, and growth [19] and is used as a biomarker of stress in humans and other vertebrates [20]. Growing hair incorporates unbound and potent cortisol molecules circulating in the bloodstream; thus, the amount of cortisol extracted from hair is commonly used to assess a long-term average of the systemic exposure to cortisol [19]. Hence, cortisol levels in hair offer a long-term measurement (e.g., spanning over weeks or months) of overall stress load, and has been used in many studies investigating chronic stress levels in a variety of mammals including grizzly bears (*Ursus arctos)*, caribou/reindeer (*Rangifer tarandus*), and owl monkeys (*Aotus nancymaae*) [21, 22, 23, 24].

Although many studies have investigated the relationship between stress levels and specific variables such as predation risk or habitat shift, there is currently limited information about the effects of landscape variables on chronic stress in wildlife (*see* [8]). To examine how chronic cortisol levels vary across a landscape requires many sampled individuals across gradients of the landscape variables of interest. Here, we explore large-scale relationships of hair cortisol levels in a solitary ungulate, moose *Alces alces*, across a 2000 km latitudinal gradient and examined how environmental factors such as long-term temperature variation, predation and inter- and intra-guild competition pressure, and anthropogenic stressors impacted hair cortisol levels in moose.

Moose are widely distributed across Sweden, thereby occurring along gradients of anthropogenic activities, carnivore distributions, climate, and sympatric ungulate species. Moose are adapted to cold climates and are thought to be especially sensitive to warm temperatures [25], which makes them a good model species for investigating temperature correlates on chronic stress levels. Additionally, declining moose numbers have been observed across the southern ranges of their distribution in North America [26, 27, 28] partly because of a variety of climate-related stressors, including higher average annual temperatures, long strings of mild winters, and increasingly favorable conditions for ticks, parasites, and other invasive species [26, 29]. Similarly, in Sweden moose in the southern portion of their range are more exposed to less favorable conditions in terms of higher temperatures and increasing prevalence of parasites [30]. Thus, we hypothesized that 1) hair cortisol levels in moose will increase along a climatic gradient from north to south because biologically moose experience increased stress at elevated temperatures which is likely to increase along the latitudinal gradient in Sweden; 2) moose calves have higher cortisol levels than adult bulls and cows likely due to elevated energy metabolism and glucocorticoids; 3) moose will experience increased stress levels in areas closer to anthropogenic centers because of disturbances associated with human activity and occupied wolf territories because wolves are one of, if not the, main predator of moose across most of their range; and 4) moose will experience increased stress levels in areas of high ungulate densities due to resource competition.

## Methods

### Study area and population

We conducted our study across Sweden where numerous climatic gradients occur from the Scandic Mountains in the west to the Baltic Sea in the east, and from the Arctic tundra in the north to the boreal and temperate broad-leaved forests in the south. Moose migrate considerable distances, from cooler summer ranges in the mountains to milder winter ranges towards the coast, compared to the south where they are more stationary [31]. In Sweden, moose are of ecological and economic importance, a national symbol that generates tourism, and Sweden’s most important game species. Moose are involved in over 10% of the wildlife-vehicle collisions [32] and their browsing on young saplings negatively impacts commercial forests.

Historically, large carnivores were abundant throughout Sweden. However, all populations were nearly extinct due to eradication campaigns ending in the 1940s. Today across Sweden, populations of brown bear *Ursus arctos* and grey wolf (*Canis lupus)* are expanding and both are regulated by licensed hunts and the removal of occasional problem animals. With populations generally stable or increasing, wolf and brown bear occur mainly in central Sweden and to the western part of the country. Sympatric ungulate species include roe deer (*Capreolus capreolus)*, domestic reindeer, fallow deer (*Dama dama)*, red deer (*Cervus elaphus)*, and wild boar (*Sus scrofa)*.

### Sample collection

**Fig 1:**
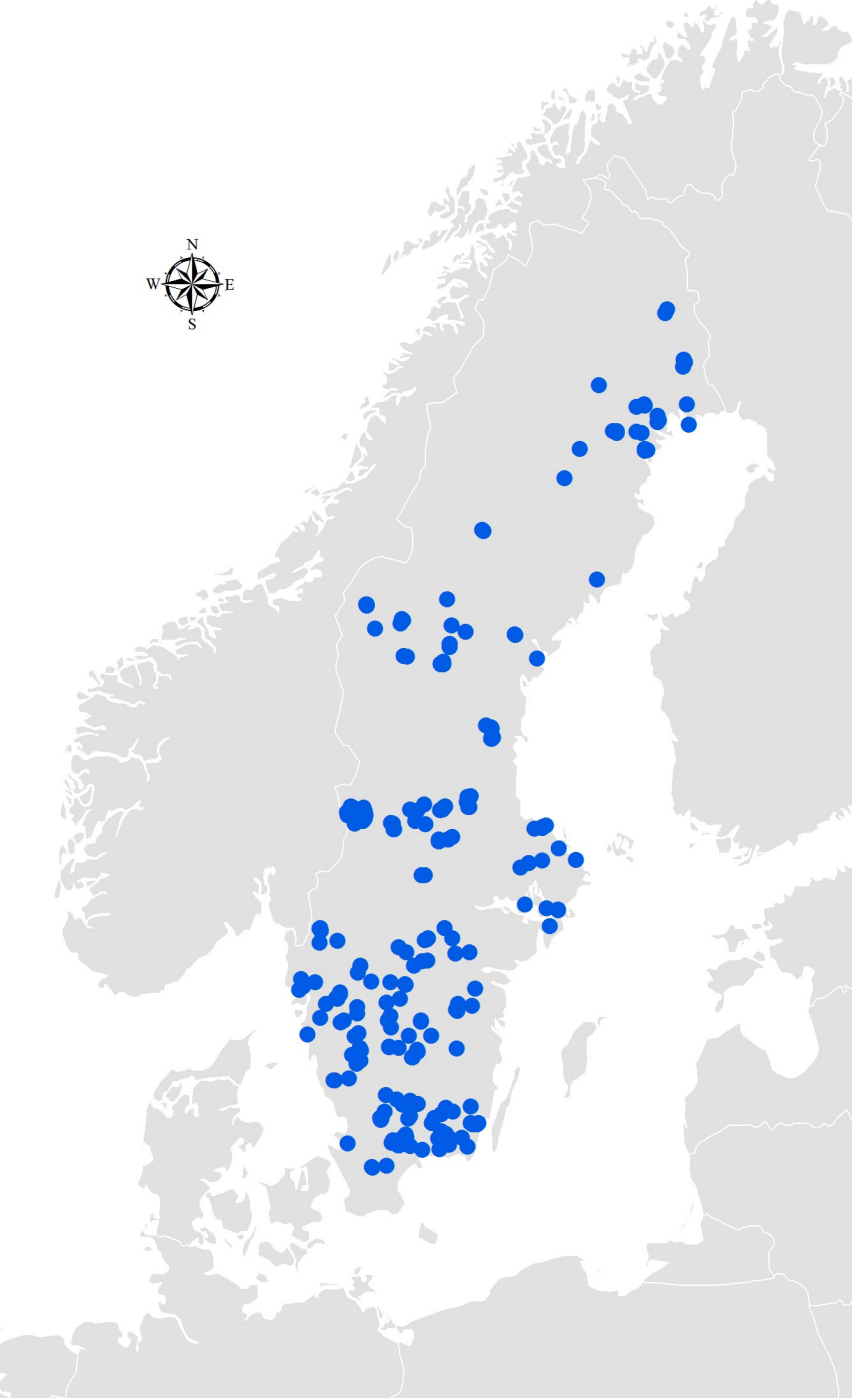
Locations of hair-sampled moose during the moose hunt, Sweden, 2012.

Hair sampling was conducted by hunters during the moose hunt in fall and winter 2012. All samples came from animals killed during the annual quota-based harvest. Because the animals were killed for non-scientific purposes, no ethical permit was required by Swedish law or by our universities. The research was reviewed and approved by our departmental animal welfare officer. Sampling protocols were distributed to hunters for hair sampling. Hair was collected from the rump by cutting the hair as close to the skin as possible using a clean knife or electric clipper over an area of approximately 4 cm^2^. Also, hunters documented the day of the hunt, GPS-location of the kill site, sex, age, reproductive status, harvest method, and general health of the moose. Because the data obtained were binary data which could easily be determined in the field, no elaborate scale was used and training was not required. Of the 1000 sample kits that were sent out to hunters, samples from a total of 389 individuals where provided by hunters nationwide (Fig 1). However, not all samples received by the lab were used in the analyses because some samples contained too few hair shafts, had hair shafts still attached to thin fragments of bloody skin, or were covered in blood.

### Hair cortisol analyses

From each of the remaining 237 samples (96 adult males, 77 adult females, 63 calves), approximately 100 mg of hair (about 20–30 hair shafts) was weighed and placed in 15 ml falcon tubes. Following the protocol developed by [21] for grizzly bear, hair samples were washed three times in methanol (99% grade chromatography methanol). During washes, 10 ml methanol was added to the falcon tube, which was slowly rotated on a rotation device for three minutes. After three minutes, methanol was removed and new methanol added. This procedure was repeated twice for every sample. After washes, hair samples were left to air dry under a fume hood (three to five days). Samples were cut with scissors into 1–2 cm long pieces and placed in a grinding jar (25 ml stainless steel grinding jars with one 20 mm stainless steel grinding ball). Hair strands were ground into a uniform hair powder using a ball mill (Retch MM 200) at 25 Hz for 3.0 min. If hair segments were present after 3.0 min processing time, grinding was continued in 15 s intervals until a uniform powder was achieved. After grinding, 50 mg of hair powder was carefully weighed out and transferred to a 1.5 ml Eppendorf tube, into which 1 ml methanol was added. After manually shaking the tube to make sure the hair powder was evenly distributed in the methanol, cortisol was extracted by rotating the tubes slowly overnight (16 hours). The morning after, samples were centrifuged for 15 min at 4600 rev/min. and 2150g, and 0.6 ml of the supernatant was transferred to a glass vial, in which it was left to evaporate at 20°C in a centrifugal evaporator. Samples were reconstituted in 0.25 ml phosphate buffer and analyzed using Neogen’s commercial enzyme-linked immunoassay kit (#402710) in duplicates. Samples were not analyzed in the order they were received by our lab, to separate samples potentially sent from the same hunter or area over several microplates. Regarding specificity of the ELISA, cross reactivity of the antibody used for the cortisol kit was (according to the manufacturer): Cortisol: 100.00%, Prednisolone: 47.42%, Cortisone: 15.77%, 11-Deoxycortisol 15.00%, Prednisone: 7.83%, Corticosterone: 4.81%, 6b-Hydro-xycortisol: 1.37%, 17-Hydroxyprogesterone: 1.36%, Deoxycorticosterone: 0.94%, Progesterone: 0.06%, Betamethasone: 0.05%, Dehydroepiandrosterone: 0.03%, Dexamethasone: 0.03%, Beclomethasone: 0.01%, d-Aldosterone: 0.01%, Testosterone: 0.01%, 17α-Hydroxypregnenolone: < 0.01%, Androstenedione: < 0.01%, Cholesterol < 0.01%, Estradiol: < 0.01%, Estriol: < 0.01%, Estrone: < 0.01%, Pregnenolone: < 0.01%. Intra- and interassay coefficients of variation (CV) were 3.9 and 15.1%, respectively. A two-fold increase in the amount of hair powder analyzed in a standard hair sample led to a following two-fold increase in detected cortisol level. The detection limit was 0.4 ng cortisol/ml (calculated by taking the average of the absorbance of the zero standard provided with the commercial kit), which corresponds to a minimum detectable quantity of approximately 0.24 pg cortisol/mg hair and is similar to what is reported in other studies (e.g. [14, 33]).

### Variables influencing long-term stress levels

We created a single candidate set (*a priori*) of linear models by grouping parameters in combinations that we predicted to be ecologically relevant for moose, including demographic group, surrounding level of anthropogenic disturbance, ungulate density (i.e., measure of competition), climate and temperature, and carnivore impact (Table 1). We included two variables to investigate impacts of anthropogenic disturbance; road density and distance to towns. Road density was quantified within a moose home range sized buffer around the kill site (1.83 km radius) in ArcGIS. Road density was calculated by measuring the total road (line) length within the buffer. Distance to towns was calculated by measuring the Euclidean distance from the kill site to the nearest settlement with >200 inhabitants. We included one climate variable (average temperature sum over the last 30 years, received from [34]) that was calculated by summing the daily average temperature for days during the growing season (> 5°C), and then creating a yearly average value for 1980–2009 (according to SMHI’s product sheet). The data was delivered as an average temperature across a sub-basin level (~500 km^2^ on average).

**Table 1:** Variables and categories included in linear model combinations to evaluate moose hair cortisol levels in Sweden, 2012.

Category	Variable	Description
Intrinsic	Demographic group	Factor with three levels (cow, bull, calf)
	Condition	Health status; factor with two levels (poor, healthy)
Anthropogenic influences	Dist. to town	Distance to towns (> 200 inhabitants)
	Road density	Road length (> 5 m wide) within a 10.5 km^2^ buffer^1^
Ungulates / competition	Ungulate	Number of ungulates shot / 1000 ha (sum of all species)
Climate and temperature	Average temp. sum	Average temperature sum (years 1980–2009)
Carnivores	Dist. to wolf	Distance to wolf territories; covariate (m)

^1^Based on an average home range size with a radius of 1.83 km (southern Sweden), see [35].

^2^Temperature based on [25].

We excluded areas far from wolf territories (> 30 km from wolf territories), and used the remaining data from this variable in our model set to analyze potential predation pressure on long-term stress levels of moose. In our model set, we included effects of human disturbance and average temperature sum (to correct for potential temperature effects).

To avoid spurious results in our models, we reduced the number of ungulate variables from five different species down to one category representing the summed densities for the different species to include their combined effect on moose hair cortisol levels. Because of the similar gradients from north to south regarding human disturbance-related variables, ungulate densities, and the climate-related variable, we expected that some variables would be correlated; therefore, we explored collinearity for parameters using Pearson’s correlation coefficient and Variance Inflation Factors (VIFs < 2 was considered acceptable; [36]).

### Candidate model set

To investigate the effects of hair cortisol levels in moose, we developed a global model from which we further developed a subset of 23 additional linear models (Gaussian distribution) with structured combinations of our variables representing the effect of carnivores as a predation stressor, ungulate density as a competition stressor, and human disturbance and our climate variable as anthropogenic stressors. In each model, we retained ‘Reproductive Status’ (calf, cow, or bull) and ‘Condition’ (healthy or poor body condition) because these were two inherently biological variables that may help explain long term variation in hair cortisol levels in the Swedish moose population. Prior to analysis, we log-transformed the response variable (hair cortisol values) to reduce the spread of the values and because cortisol values are normally sparse [37], and scaled to a mean of zero and unit variance all of our continuous landscape level variables to allow for comparability in the estimates of their effect sizes. We used the *margins* and *ggpredict* packages in R to estimate the average marginal effect for any significant variables in our model(s).

We compared linear models based on differences in Akaike's information criterion corrected for small sample size (ΔAIC_c_) to assess model weights, and ranked candidate models using ΔAICc [38]. We used Akaike weights to determine the relative support for a model, and used model averaging from all model combinations across parameters and calculated unconditional variance estimates and associated 95% confidence intervals. Further, we determined if our covariates had influence on hair cortisol levels by examining if the confidence intervals overlapped zero.

## Results

**Fig 2:**
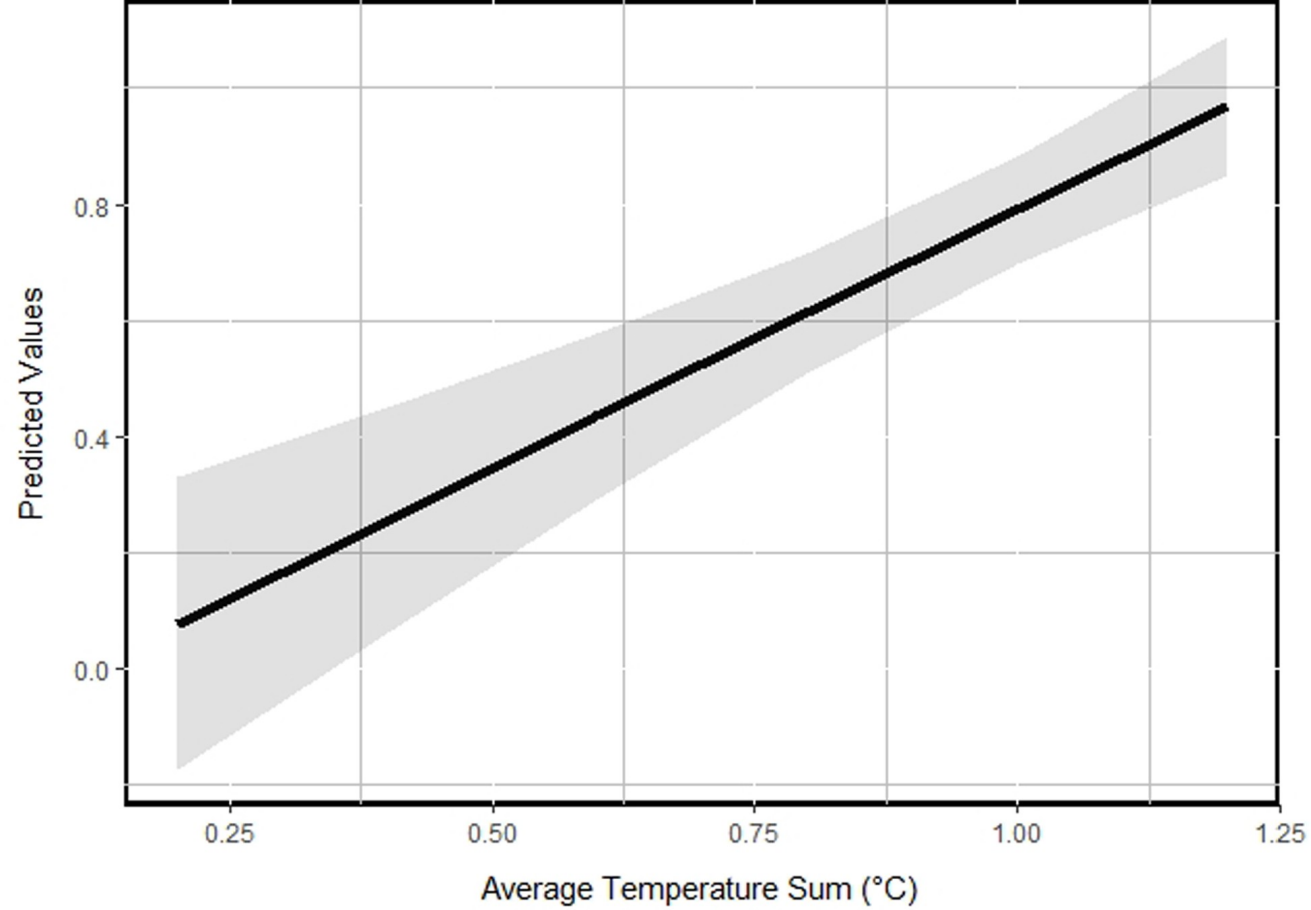
Mean fitted (predictive) values for the marginal effect of hair cortisol levels on the long-term average temperature sum in the area where they lived, Sweden, 2012.

**Fig 3:**
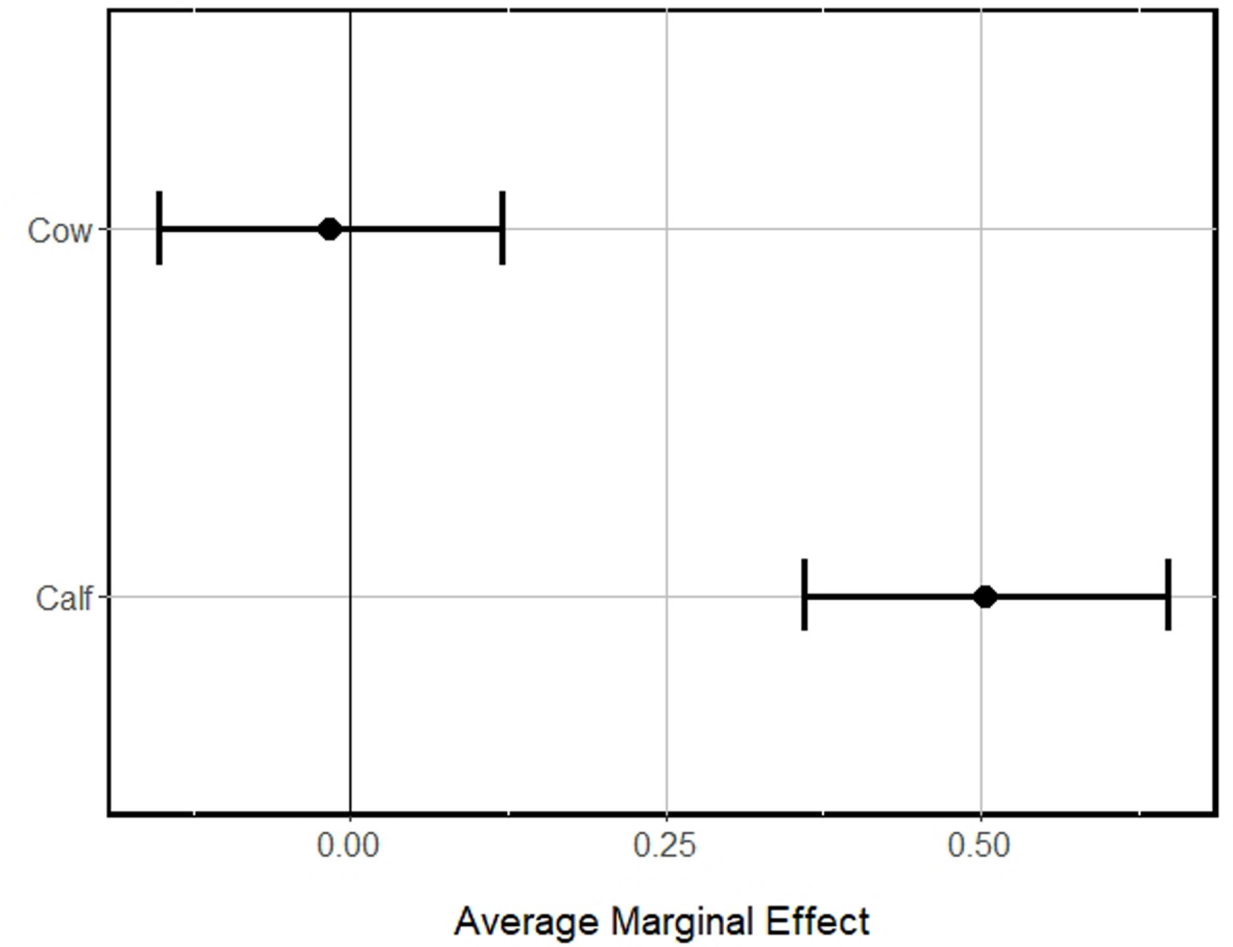
Mean fitted values of hair cortisol levels for the marginal effect of cow and calf moose in Sweden, 2012.

**Fig 4:**
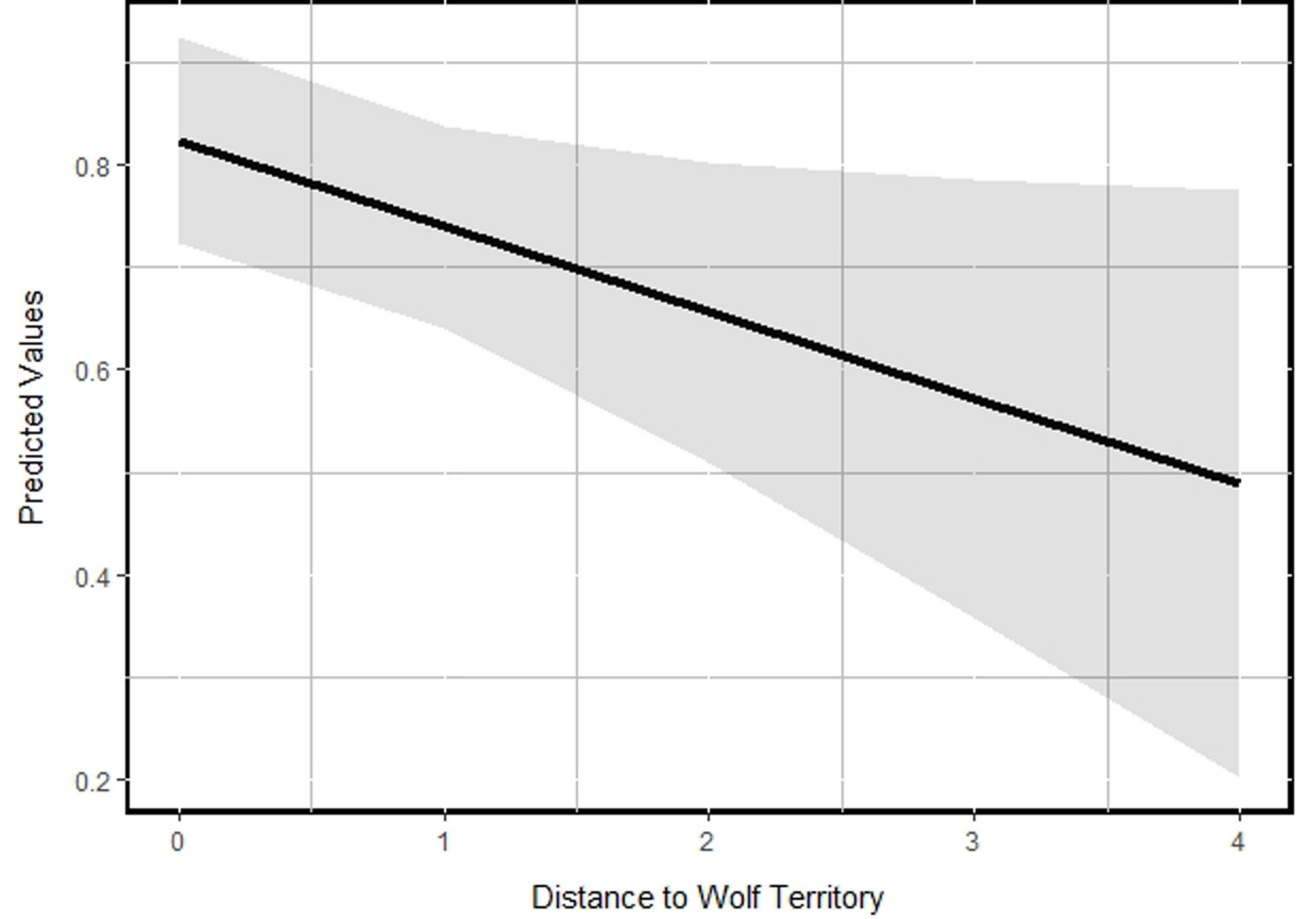
Mean fitted (predictive) values for the marginal effect of hair cortisol levels on distance to wolf territory of moose in Sweden, 2012.

During the fall and winter of 2012, we collected hair samples from 237 hunter harvested moose carcasses (96 adult males, 77 adult females, 63 calves). Initial removal of missing body ‘Condition’ values reduced our sample size to 232 (93 adult males, 77 adult females, 62 calves). On average, hair cortisol levels for bull, cow, and calf moose were 2.42 (*SE* = 0.13), 2.49 (*SE* = 0.16), and 4.09 (*SE* = 0.28), respectively. Our top model (~ Dem. Group + Condition + Avg. Temp Sum + Wolf) was supported with 37% of the overall model weight, thus our approach to model average our beta coefficients was warranted (Table 2). We determined that hair cortisol levels in moose were positively related to the climatic gradient in Sweden (β_Avg. Temperature Sum_ = 0.9136, 95% *CI* = 0.5555–1.2716), suggesting that moose in warmer regions generally had higher hair cortisol levels than moose in colder regions (Table 3, Fig 2). Additionally, we detected support for differences in ‘demographic group’ indicating that moose calves had substantially higher cortisol levels than adult bulls and cows (β_Dem. Group (Calf)_ = 0.5002, 95% *CI* = 0.3539–0.6465; Fig 3). Lastly, we noted the distance to an occupied wolf territory was inversely related to long term stress levels in moose (β_Distance to Wolf_ = -0.0846, 95% *CI* = -0.1636 - -0.0054; Fig 4). There was no effect of the condition of the moose (‘poor’ versus ‘healthy; β_Condition (Poor)_ = 0.2695, 95% *CI* = -0.0067–0.5457), the density of ungulates (i.e., competition) within the home range buffer where the moose was harvested (β_Ungulate Density_ = 0.0289, 95% *CI* = -0.1126–0.1704), whether the moose was located close to town or not (β_Distance to Town_ = 0.0066, 95% *CI* = -0.1212–0.1343), or the level of road density within the home range buffer where the moose was harvested (β_Road Density_ = 0.0140, 95% *CI* = -0.0648–0.0929), respectively, on the long term hair cortisol levels in moose in Sweden (Table 3).

**Table 2:** Variables included in the top 9 comprehensive linear models (within ΔAIC < 6; for reference) to evaluate hair cortisol levels in moose sampled across the Swedish distribution in 2012.

	*K*	AICc	ΔAICc	AICc Weight	Model Likelihood	LL	Cum. Weight
Dem. Group + Condition + Avg. Temp Sum + Wolf	7	292.5	0.00	0.37	1.00	-139.00	0.37
Dem. Group + Condition + Roads + Avg. Temp Sum + Wolf	8	294.46	1.96	0.14	0.38	-138.91	0.51
Dem. Group + Condition + Town + Avg. Temp Sum + Wolf	8	294.64	2.13	0.13	0.34	-138.99	0.64
Dem. Group + Condition + Avg. Temp Sum	6	294.84	2.33	0.12	0.31	-141.23	0.76
Dem. Group + Condition + Roads + Town + Avg. Temp Sum + Wolf	9	296.59	4.09	0.05	0.13	-138.89	0.81
Dem. Group + Condition + Avg. Temp Sum + Ungulate	7	296.85	4.35	0.04	0.11	-141.18	0.85
Dem. Group + Condition + Roads + Avg. Temp Sum	7	296.95	4.45	0.04	0.11	-141.23	0.89
Dem. Group + Condition + Town + Avg. Temp Sum	7	296.96	4.46	0.04	0.11	-141.23	0.93
*Global Model	10	298.37	5.87	0.02	0.05	-138.69	0.95

*Global model = Dem. Group + Condition + Roads + Town + Avg. Temp Sum + Wolf + Ungulate

**Table 3:** Model-average parameter estimates (β), standard errors (se), test statistics (t-values), p-values, and confidence intervals for variables in the linear models to evaluate hair cortisol levels in moose across the Swedish distribution in 2012. The reference levels for comparison for the categorical variables ‘Demographic group’ and ‘Condition’, are ‘Bull’ and ‘Healthy’, respectively.

					95%	
	β	se	z value	Pr (>|z|)	LCL	UCL
(Intercept)	-0.0874	0.2000	0.437	0.6621	-0.4794	0.3046
Demographic group						
Calf	0.5002	0.0746	6.702	<0.0002	0.3539	0.6465
Cow	-0.0218	0.0705	0.309	0.7572	-0.1599	0.1163
Condition (Poor)	0.2695	0.1409	1.912	0.0559	-0.0067	0.5457
Average temperature sum	0.9136	0.1826	5.001	<0.0006	0.5555	1.2716
Distance to wolf	-0.0846	0.0404	2.094	0.0362	-0.1636	-0.0054
Road density	0.0140	0.0402	0.348	0.7276	-0.0648	0.0929
Distance to town	0.0066	0.0652	0.101	0.9196	-0.1212	0.1343
Ungulate density	0.0289	0.0722	0.401	0.6887	-0.1126	0.1704

## Discussion

Our study demonstrated that moose hair cortisol levels are not uniform across the Swedish landscape. As predicted by our first hypothesis, there was a clear gradient in cortisol levels from north to south, with moose having higher levels in the south, and our analyses suggest the climatic gradient (i.e., average temperature sum) was an important predictor of stress levels in moose. [25] noted that moose decreased their activity at a temperature of 14°C. At 20°C moose were open-mouth panting and substantially reduced movement. Biologically, moose respond to heat stress with increased respiration rates, decreased food intake and increased water intake [25]. When temperatures rise, moose increase activities that cool their bodies (e.g., wading in lakes or lying in swamps) while reducing heat-producing activities such as walking and eating. Thus, hair cortisol levels could be compounded by moose movement (and stress levels) which may be substantially affected by increasing temperature [39]. Moreover, warmer temperatures increase prevalence of pathogens and parasites, which are more abundant in the southern range [30]. Further, moose in poor condition are more heat sensitive than healthy moose, which suggests that warming temperatures may affect health-compromised individuals more negatively than healthy individuals [40]. If there is indeed a relationship between warming temperature and moose health, we may see higher moose mortality and disease rates in the future, especially in the southern ranges, and especially in young moose.

We also found support for our second hypothesis that calves had higher hair cortisol levels than adults. In ungulates, as well as in other mammals, younger individuals typically have higher cortisol levels than adults [41, 42]. The higher levels of hair cortisol in calves are likely due to elevated energy metabolism and glucocorticoids [43]. Further, moose had higher stress levels in the south including higher presence of embryonic mortality, and high prevalence of a tick-borne pathogen that may affect moose calf health in the future [44].

For hypothesis three, we determined that moose hair cortisol levels were higher the closer moose were to occupied wolf territories, and areas with higher wolf occurrence are generally characterized by having less human activity (fewer roads, towns, and human inhabitants). Although we did not detect the anthropogenic variables (i.e., distance to town and the densities of roads) to be important in our model, it is possible that risk and human activity are working at different scales than we measured. Further, we did not account for different road or town sizes and it is likely that areas with high carnivore density have fewer large roads or towns [45]. Indeed, our data showed that areas with higher wolf occurrence had fewer roads and were situated farther from towns. Also, it is possible these areas are less fragmented, which may mean lower ambient temperatures during hot days, as large tracts of mature forest are important locally for thermoregulation [46]. Nevertheless, the closer moose were to occupied wolf territories the higher the cortisol levels which is not surprising because wolves, where they occur, are the main predator of moose [29, 47].

There was no support for our last hypothesis that competitor density causes higher cortisol levels. Other research has suggested diet displacement of moose by red deer [48]. But perhaps our estimates of ungulate densities were to crude to detect such an effect or simply because such competition does not easily lead to elevated cortisol levels.

To conclude, temperature, distance to occupied wolf territories, and reproductive status (i.e., calves) were the most important factors explaining the variation in stress hormone levels in moose. The average temperature is predicted to increase by 1.4–5.8°C over the next 100 years [49] and moose are behaviorally, physiologically, and morphologically adapted to cold environments [50] therefore, higher temperatures may ultimately affect moose health and distribution in the future [28].

The short term solution to lessen potential impacts of warming temperatures and human activity on moose and other heat-sensitive wildlife species may be to conserve continuous forests, which increase connectivity and genetic variability for populations and provide cooler temperatures along with low human activity. In particular, mature forests may provide important areas for thermoregulation at local scales [51]. Hence, continuous areas with mature forests may become increasingly important for moose and other wildlife species.

## Supporting information

S1 Data (TXT)
